# The importance of cytoplasmic strings during early human embryonic development

**DOI:** 10.3389/fcell.2023.1177279

**Published:** 2023-07-11

**Authors:** Kata Joo, Annamaria Nemes, Beata Dudas, Eva Berkes-Bara, Akos Murber, Janos Urbancsek, Peter Fancsovits

**Affiliations:** Division of Assisted Reproduction, Department of Obstetrics and Gynaecology, Semmelweis University, Budapest, Hungary

**Keywords:** human *in vitro* fertilisation, embryo quality, cytoplasmic strings, time lapse, embryo development, human blastocyst, Intelligent Data Analysis Score, Known Implantation Data Score

## Abstract

**Objectives:** During human *in vitro* fertilisation (IVF) treatments, embryologists attempt to select the most viable embryos for embryo transfer (ET). Previously, embryos were evaluated based on light microscopic morphological parameters. However, this is currently accomplished by morphokinetic analysis of time-lapse recordings. This technique provides us the opportunity to observe cytoplasmic strings at the blastocyst stage. The aim of this work was to examine the relationship between the presence of cytoplasmic strings (CS) and the embryo viability in human *in vitro* fertilised embryos.

**Study design:** Herein, we present an evaluation of the morphokinetic data on the development of embryos obtained during IVF treatments performed at the Division of Assisted Reproduction between December 2020 and March 2021. The dynamics of embryo development, embryo morphology, and morphokinetic scores generated by a time-lapse system were compared between the presence of cytoplasmic strings (CS+) and their absence (CS-) at the blastocyst stage.

**Results:** The development of 208 embryos from 78 patients was examined. Moreover, 81.2% of the embryos had CS in the blastocyst stage; 77% of CS existed in embryos created by conventional IVF, while 86% of CS existed in embryos fertilised by intracytoplasmic sperm injection (ICSI) (*p* = 0.08). A greater number of CS+ embryos developed into a higher quality blastocyst (52.1% vs. 20.5%, *p* = 0.02). The morphokinetic score values characterising the development of embryos, such as Known Implantation Data Score (KIDScore) and Intelligent Data Analysis (iDAScore), were higher in CS+ groups (KID: 6.1 ± 2.1 vs. 4.7 ± 2.07; iDA: 8.0 ± 1.9 vs. 6.8 ± 2.3, *p* < 0.01). The dynamics of the early embryo development were similar between the two groups; however, CS+ embryos reached the blastocyst stage significantly earlier (tB: 103.9 h vs. tB: 107.6 h; *p* = 0.001).

**Conclusion:** Based on our results, the number of embryos with cytoplasmic strings was higher than that without cytoplasmic strings, and its presence is not related to the fertilisation method. These embryos reached the blastocyst stage earlier, and their morphokinetic (KIDScore and iDAScore) parameters were better. All these results suggest that the presence of CS indicates higher embryo viability. The examination of this feature may help us make decisions about the embryos with higher implantation potential.

## Introduction

During *in vitro* fertilisation (IVF-ET) treatments, embryologists attempt to select the best developing and most viable embryo for embryo transfer (ET). Previously, conventional embryo selection methods were based on morphology grading systems. In addition to the traditional morphological grading techniques (Gardner & Schoolcraft, 1999; Alpha Scientists in Reproductive Medicine and ESHRE Special Interest Group of Embryology, 2011; Prados et al., 2012), the morphokinetic evaluation of embryo development by time-lapse recordings commenced in practice (Meseguer et al., 2012). Conventional embryo culture and traditional morphological evaluations were performed at static time points, which has a limited ability to predict the most viable embryo (Guerif et al., 2007), since they provide a “snap-shot” of the embryo development.

The appearance and clinical application of the time-lapse system create new opportunities for the detailed evaluation of embryo morphology. The system records embryo development at regular intervals of 5–15 min (Rubio et al., 2014).

Embryo development can be analysed by several morphology evaluation programs. The KIDScore decision support tool (Vitrolife, Göteborg, Sweden) is based on the known implantation data of the world’s largest embryo development database, and it provides a morphokinetic score to annotated embryos. Those embryos, which ranked low by the tool, have statistically lower chances of implantation (Berntsen et al., 2022). Intelligent Data Analysis Score (iDAScore) (Vitrolife) () is a grading system with artificial intelligence. This artificial intelligent system compares the examined embryo with a similar developmental pattern. The system has the ability to recognise the developmental stage and morphology of embryos without any human interaction and generate a score for the possibility of implantation (Kragh et al., 2019; Ueno et al., 2021).

Literature has heavily focused on the prediction of morphokinetic-based embryo assessment and implantation and pregnancy outcomes during the previous years. Furthermore, the analysis of the time-lapse videos made it possible to identify and observe the behaviour of dynamically changing structures like cytoplasmic strings. This observation is impossible during the conventional microscopic morphology assessment.

In addition to the frequent presence of cytoplasmic strings in different cell functions, we have limited information about their function in human embryos. The observation of morphology and behaviour of these cytoplasmic strings can aid us to reach a closer understanding of the early embryonic development.

In the early 2000s, the occurrence of cytoplasmic strings at the blastocyst stage was classified as a negative sign of embryo viability (Scott, 2000), but more recently, it has been described as a positive feature (The ESHRE Capri Workshop Group, 2010; Ebner et al., 2020; Ma, Jin, and Huang, 2020; The ESHRE Capri Workshop Group, 2020; Eastick et al., 2021). However, the importance and clinical significance of this structure are still controversial. Previous proposals suggested that signalling molecules could migrate through cytoplasmic strings by vesicle transport or via cytoplasmic material exchange (Gustafson and Wolpert, 1961; Miller et al., 1995; Salas-Vidal and Lomelí, 2004). The authors observed retrograde transport, but it is still not known exactly what molecules are required for the transport of particles through the cytoplasmic strings.

The aim of our study was to investigate the occurrence and morphological characteristics of cytoplasmic strings in the human blastocyst and to examine the relationship between the occurrence of strings and embryo viability during human blastocyst formation.

## Materials and methods

### Study design

**FIGURE 1:**
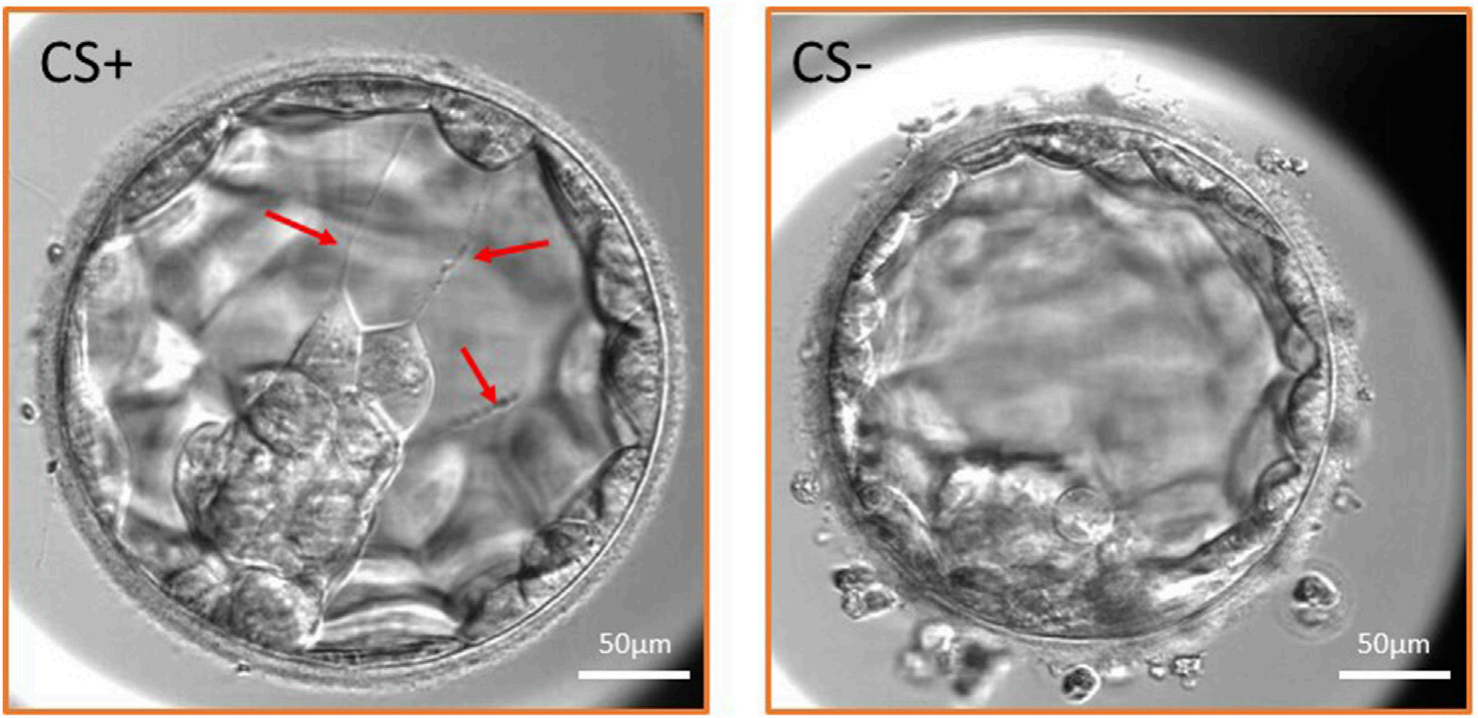
Expanded blastocyst with cytoplasmic strings (CS+ group) and without CS (CS- group). Three cytoplasmic strings are shown in the CS+ embryo traversing the blastocoel cavity (red arrows) and maintaining connection between the inner cell mass and the mural trophectoderm cells.

This retrospective study was conducted at the Division of Assisted Reproduction, Department of Obstetrics and Gynaecology, Semmelweis University, Budapest, Hungary. A total of 78 IVF treatment (IVF-ET) cycles performed routinely due to infertility at our department between December 2020 and March 2021 were included in the study. The exclusion criteria did not include maternal age, male infertility parameter, method of fertilisation, BMI, and treatment indication. Those IVF treatment cycles were examined to identify at least one normally fertilised (two pronuclei) oocyte that developed into blastocyst by day 5. More embryos of the same IVF-ET cycle were examined to analyse the development of 208 embryos which reached the expanded blastocyst stage until the ET. Blastocyst development was analysed in detail, particularly the occurrence and morphology of cytoplasmic strings. Embryo development and implantation were compared between blastocysts containing cytoplasmic strings (CS+ group) and blastocysts without CS (CS- group) (Figure 1).

### Ovarian stimulation and oocyte collection

A gonadotropin ovarian stimulation protocol with gonadotropin-releasing hormone (GnRH) antagonist was used to achieve multiple follicular growth. Human menopausal gonadotropin (hMG) (Menopur, Parsippany, New Jersey; Ferring, Saint-Prex, Switzerland) or follicular stimulating hormone (FSH) (Fostimon HP, IBSA, Pambio Noranco, Switzerland) was used for ovarian stimulation, which was monitored by estradiol measurements and transvaginal ultrasound examination on alternate days. A dose of 0.25 mg/day cetrorelix (Cetrotide; Serono, Rome, Italy) was administered from the day of ovarian stimulation where the diameter of the leading follicle was ≥14 mm. Ovulation was induced with 5,000 IU of human chorionic gonadotropin (hCG) (Ovitrelle; Serono, Geneva, Switzerland) when at least one follicle with a diameter of 18 mm and three or more follicles with a diameter of 16 mm were seen on ultrasound and serum estradiol levels reached 2–300 pg/mL per ≥16 mm follicle. Transvaginal ultrasound-guided aspiration of follicles was performed 36 h after hCG administration. Oocytes were collected from the follicular fluid and cultured at 37°C and 6% CO_2_ and 5% O_2_ levels until fertilisation.

### Sperm preparation and fertilisation

All laboratory procedures were performed using the standard protocol (Fancsovits et al., 2015). Oocyte and embryo culture was performed in a culture media product line called “G-series” produced by Vitrolife. A semen sample was prepared for fertilisation by density gradient centrifugation followed by the swim-up technique to isolate progressive motile sperms. Two-layer density gradient centrifugation (90% and 45% SpermGrade (Vitrolife) solution) was performed for the separation of motile sperm cells from the semen sample. Samples were centrifuged at 400 g for 20 min (Centrifuge 5702; Eppendorf, Hamburg, Germany). Viable sperms gathered at the bottom of the centrifuge tube were washed two times with warm sperm preparation media (G-MOPS+; Vitrolife) at 400 g for 10 min. The sperm fraction was resuspended with 0.1–0.5 mL fertilisation media (G-IVF^+^; Vitrolife) according to the semen quality. Sperm concentration and motility were counted again after sperm preparation.

Fertilisation was carried out by conventional IVF or intracytoplasmic sperm injection (ICSI), depending on the patient’s history and the semen quality. In case of conventional IVF, a maximum of five cumulus–oocyte complexes/well in a four-well culture dish (NUNC; Thermo Fisher, Waltham, Massachusetts) were co-incubated with 3 × 10^5^ progressive motile sperms/mL in G-IVF+ (Vitrolife) culture fertilisation media.

During ICSI treatment, oocytes were injected following enzymatic removal of cumulus cells using hyaluronidase solution. Fertilisation by injecting a single sperm cell into the oocyte is recommended, if andrological parameters or previous fertilisation rate justifies the treatment. Intracytoplasmic sperm injection (ICSI) was performed 4–6 h after oocyte retrieval (Ebner et al., 2001). Only mature oocytes with a visible first polar body were injected. Oocytes were placed into a EmbryoSlide^+^ time-lapse dish (Vitrolife) containing embryo culture media (G-TL; Vitrolife) and inserted into the EmbryoScope^+^ time-lapse incubator (Vitrolife) after sperm injection.

Zygotes were assessed for signs of fertilisation 16–18 h after insemination and were considered normally fertilised if two pronuclei were clearly visible.

### Embryo culture and embryo transfer

**FIGURE 2:**
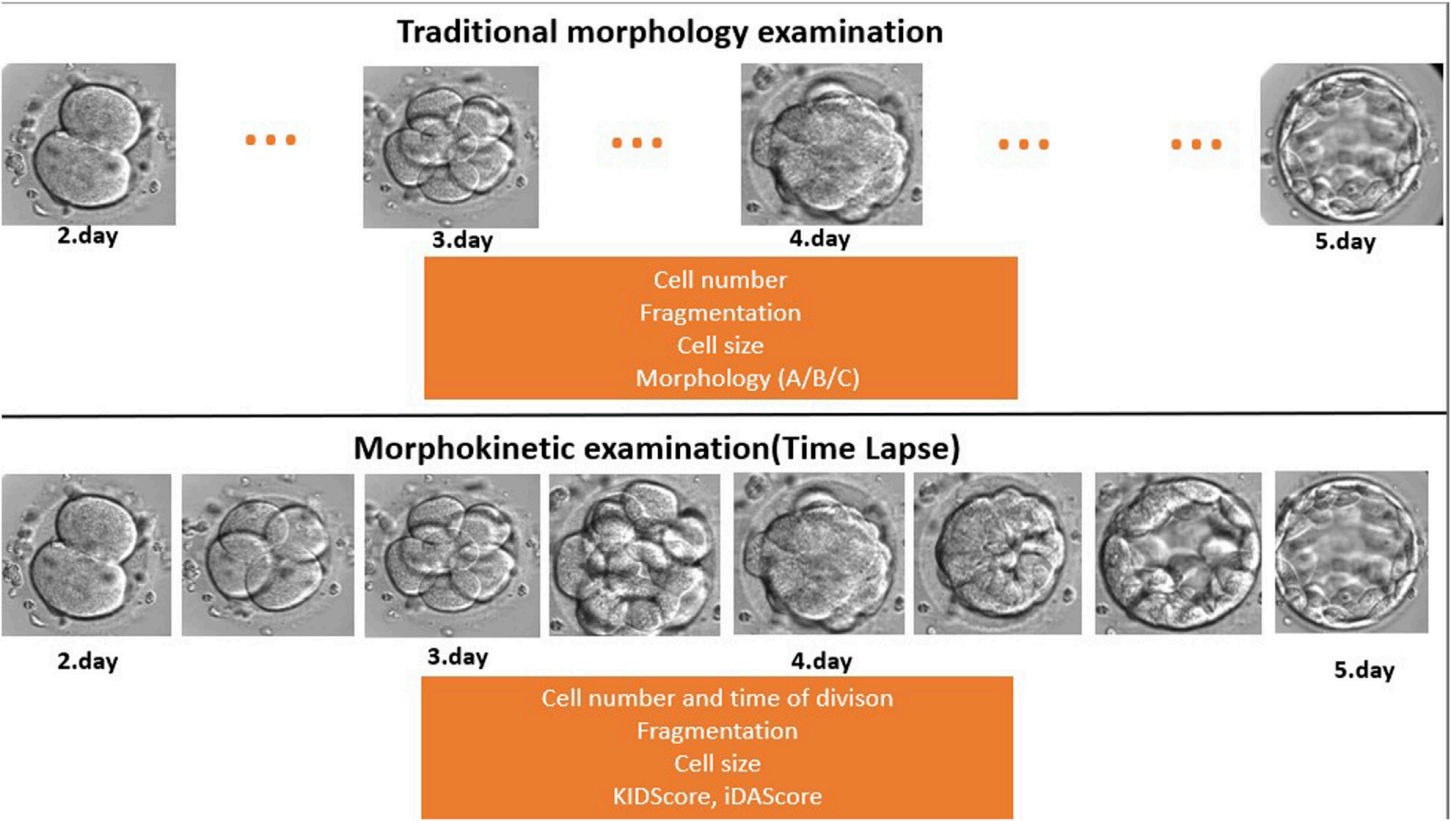
Traditional morphology examination compared to time-lapse-based morphokinetic examination. During the traditional microscopic evaluation, the embryologists miss developmental events and stages because they check the embryos once a day (dots … mean the missed/not seen developmental stage). On the other hand, the time-lapse system makes a recording every 10 min, which provides a continuous follow-up.

Embryos were cultured for at least 5 days in the EmbryoScope^+^ time-lapse incubator in EmbryoSlide^+^ dishes at 37°C under 6% CO_2_ and 5% O_2_ conditions, according to the manufacturer’s instruction. This culture technique was carried out in a microwell culture dish, which contains 8 or 16 microwells. The application of this embryo culture technique made it possible to monitor the full course of embryo development under undisturbed culture conditions without removing the embryos from the safe environment of the time-lapse incubator (Figure 2). Embryo development and morphology were evaluated by the analysis of time-lapse videos each day by embryologists (Alpha Scientists in Reproductive Medicine and ESHRE Special Interest Group of Embryology, 2011; Kovacs et al., 2019), according to standard criteria.

Embryos at an optimal developmental stage and of highest morphology grade were selected for ET 5 days after fertilisation. One or two embryos were transferred at a time depending on the female patient’s age and history. The transfer media used were the EmbryoGlue (Vitrolife). Wallace (SIMS Portex Ltd., Målov, Denmark) or Tight Difficult Transfer (TDT) catheters (Laboratoire CCD, Paris, France) were used for the ET. Non-transferred embryos, which were considered showing the normal blastocyst development and morphology according to the Gardner’s criteria (Gardner and Schoolcraft, 1999), were vitrified and stored in liquid nitrogen according to the patient’s request.

Pregnancy was confirmed by the presence of a gestational sac with foetal heart activity 4 weeks after ET. The implantation rate was calculated by the number of gestational sacs with foetal heart activity divided by the number of transferred embryos. When calculating the implantation rate, only embryos with known implantation data were taken into account. This means that those cycles were excluded where two embryos were transferred at the same time but only one implanted. Since it was not possible to determine which embryo was implanted, data on mixed embryo transfer, when CS+ and CS- embryos were transferred and implanted together, were also excluded.

### Assessment of embryo morphology

Morphology and kinetics of embryo development were also monitored and analysed using EmbryoViewer (version: 7.8.4.28428; Vitrolife) and iDAScore (version: 1.2.0.0.; Vitrolife) software. The time of the pronuclear fading (tPNF); 2-cell (t2), 3-cell (t3), 4-cell (t4), 5-cell (t5), and 8-cell (t8) stages; end of compaction (tM); initiation of blastocyst formation (tsB); and full blastocyst stage (tB) was recorded. The fragmentation rate; morphology at 4- and 8-cell and blastocyst stages; and the presence of cytoplasmic strings were also observed.

Detailed evaluation of the embryo morphology was performed on the third and fifth days of embryo development, without removing the culture dishes from the incubator. The morphology grade and amount of fragmentation were recorded at the 8-cell stage on day 3 (48 h post-insemination). The developmental stage and the morphology of the trophectoderm layer (TE) and inner cell mass (ICM) were recorded according to Gardner’s grading system on day 5 (112–114 h post-insemination) (Gardner and Schoolcraft, 1999).

Embryos with stage-appropriate cell sizes and low fragmentation were graded as good quality embryos on day 3. Embryos on day 5 were considered of good quality, if they reached at least the blastocyst stage with grade A or B TE and grade A or B ICM (Alpha Scientists in Reproductive Medicine and ESHRE Special Interest Group of Embryology, 2011). KIDScore and iDAScore were also calculated for each blastocyst on day 5.

A cytoplasmic string assessment was performed after ET and vitrification. The time-lapse videos and their images were analysed in 11 focal planes. The annotation was carried out by one embryologist, who has experience in time-lapse embryo annotation, to minimise personal bias. We determined the frequency of occurrence of cytoplasmic strings and their relation to the female age and the method of fertilisation. The dynamics of embryo development, embryo morphology at the 8-cell stage and at the blastocyst stage, KIDScore, iDAScore, and the implantation rate were compared between CS+ and CS- groups.

### Statistical analysis

All statistical analyses were performed using Statistica software (TIBCO Software Inc.). Continuous variables were presented as mean and standard deviation, and proportional variables were presented as percentages. The Mann–Whitney *U*-test was used to compare mean values, while chi-squared and Fisher tests were used to compare proportional values. Statistical significance was set at *p* < 0.05.

## Results

### The appearance of cytoplasmic strings

**FIGURE 3:**
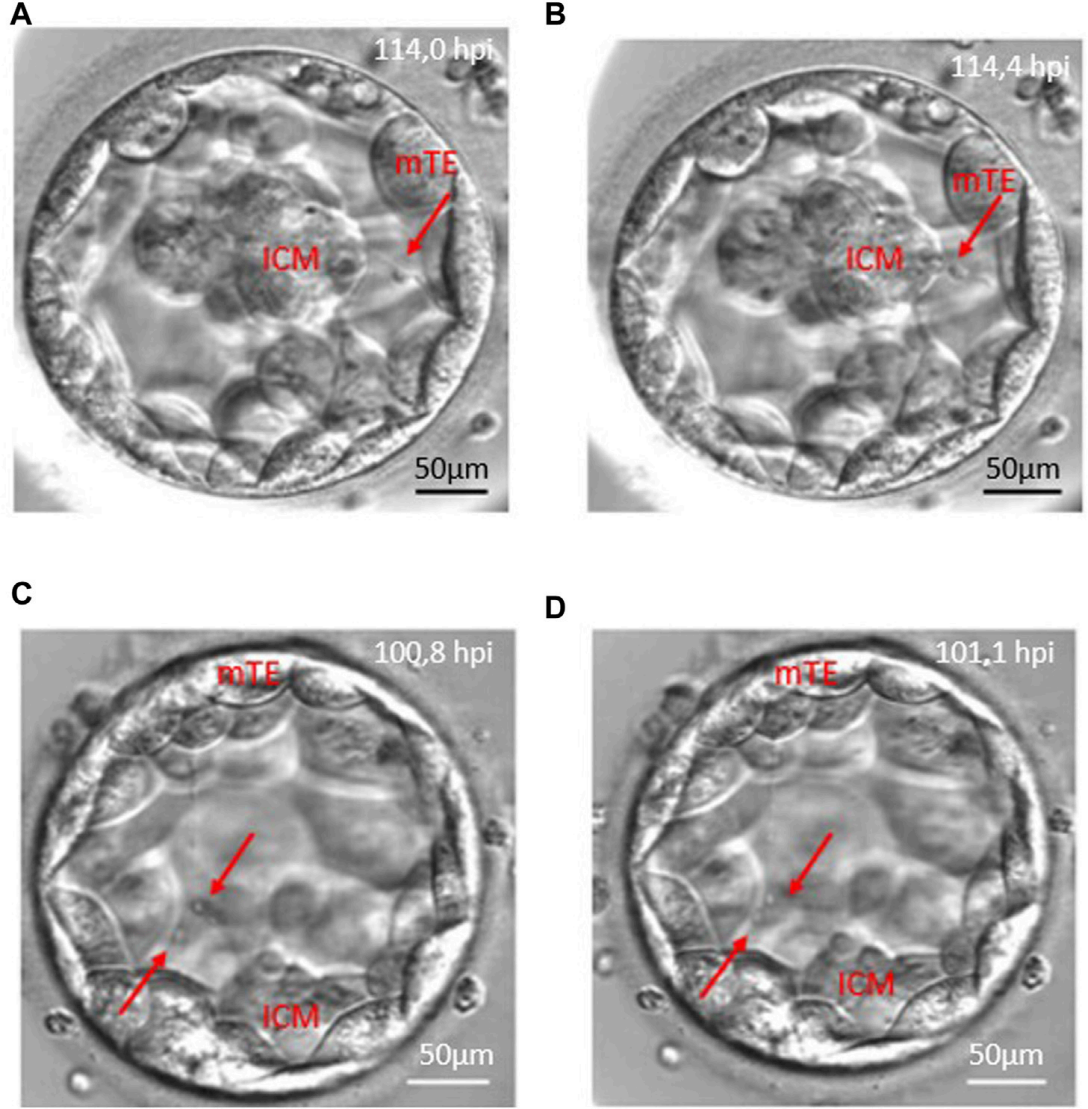
Anterograde and retrograde transport along the cytoplasmic string. Red arrows point to vesicle-like structures moving along cytoplasmic strings. Panels **(A, B)** demonstrate the retrograde transport which starts from the mural trophoblast (mTE) to the inner cell mass (ICM). Panels **(C, D)** show an anterograde transport from the inner cell mass (ICM) to the mural trophoblast (mTE) moving of two vesicles.

Of the total of 498 retrieved oocytes, 354 zygotes showed normal fertilisation (two pronuclei), and 208 embryos reached the blastocyst stage (58.8%) until the fifth day during the study period. Detailed morphokinetic analysis was performed on the embryos, which fulfilled our criteria. According to our observations, the long traversing cytoplasmic strings were visible during blastocyst formation and the full blastocyst stage. Of the 169 CS+ blastocysts, there were 135 cases (79.8%) where the string was single-ending (non-branching) and 34 cases (20.1%) where the string was multiple-ending (branching). In 128 blastocysts (75.7%), the cytoplasmic strings were thin, while 41 blastocysts (24.3%) contained thick strings. There were single- and multiple-stranded forms, and they showed considerable variation in thickness. The CS always maintain connection between the ICM and mural TE cells, and there might be a transport along the strings as shown in Figure 3 with red arrows (Figure 3).

The analysis of the CS+ embryo videos suggests that there is a transport along the cytoplasmic string that moves in both directions, anterograde and retrograde, related to the ICM, or the migration could be bidirectional (Figure 3; Supplementary Video S1).

### Development of CS+ and CS- embryos

Cytoplasmic strings can be observed from the expansion of the blastocyst. All the blastocysts, developed from the normally fertilised zygote, were examined, and 81% contained cytoplasmic strings, while 19% of the embryos did not contain cytoplasmic strings. The rate of CS+ embryos was 77% in conventional IVF and 86% in ICSI cycles. The difference between the two fertilisation methods was not significant (*p* = 0.08). The presence of cytoplasmic strings is not related to the maternal age (CS+: 35.7 ± 4.6 years; CS-:35.8 ± 4.04 years, *p* = 0.89).

The dynamics of embryo development were similar between CS+ and CS- groups until the compaction and morula stages. However, as shown in Table 1, CS+ embryos began blastocyst formation earlier and took, on average, 3 h less time than CS- embryos to reach the entire blastocyst stage (Table 1.).

**TABLE 1:** Kinetics of embryo development in cytoplasmic string containing (CS+) and not containing (CS-) groups.

	CS+ (hour)	CS-(hour)	*p*-value
tPNf	22.9 ± 3.1	23.3 ± 2.7	0.48
t2	25.5 ± 3.2	25.8 ± 2.8	0.56
t3	34.9 ± 4.8	35.2 ± 4.7	0.69
t4	36.9 ± 4.5	36.1 ± 4.9	0.69
t5	46.7 ± 8.2	47.1 ± 8.2	0.72
t8	55.8 ± 8.2	57.2 ± 9.4	0.51
tM	85.2 ± 7.6	85.2 ± 7.4	0.93
tsB	96.2 ± 6.8	99.2 ± 6.4	<0.01
tB	103.9 ± 7.7	107.6 ± 7.0	<0.01

Data are mean ± SD.

tPnf: time of pronuclei fading.

t2: time to the 2-cell stage.

t3: time to the 3-cell stage.

t4: time to the 4-cell stage.

t5: time to the 5-cell stage.

t8: time to the 8-cell stage.

tM: time of end of compaction.

tsB: time of initiation of blastulation.

tB: time to full blastocyst stage.

In both the CS+ and CS- groups, the proportion of good quality embryos at the 8-cell stage was similar. However, significantly more CS+ embryos developed into good quality blastocysts (AA, AB, BB, and BA) compared to CS- embryos. KIDScore and iDAScore were significantly higher in CS+ blastocysts than CS- blastocysts, as shown in Table 2.

**TABLE 2:** Embryo quality and morphokinetic scores in cytoplasmic string containing (CS+) and not containing (CS-) groups.

	CS+	CS-	*p*-value
Number of embryos	169 (81.2%)	39 (18.8%)	
Good quality 8-cell stage embryo	58 (34.3%)	10 (25.6%)	0.30
Good quality blastocyst (AA, AB, BA, and BB)	88 (52.1%)	8 (20.5%)	0.02
KIDScore^a^	6.1 ± 2.1	4.7 ± 2.1	<0.01
iDAScore^a^	8.0 ± 1.9	6.8 ± 2.3	<0.01
Implantation rate	25/63 ET (39.7%)	1/7 ET (14.3%)	0.19

^a^ Values are mean ± SD.

CS+: blastocyst with cytoplasmic strings.

CS-: blastocyst without cytoplasmic strings.

KIDScore: Known Implantation Data Score.

iDAScore: Intelligent Data Analysis Score.

Out of the 208 analysed embryos, 83 were transferred. However, only 70 embryos with known implantation data were used for the calculation of implantation. The implantation rate was 39.7% in the CS+ group and 14.3% in the CS- group, but this difference did not reach the level of significance (*p* = 0.16).

## Discussion

Investigation of the presence and function of cytoplasmic strings in the human blastocyst is a controversial topic among embryologists (Scott, 2000; Hardarson, Van Landuyt, and Jones, 2012; Ebner et al., 2020; Ma, Jin, and Huang, 2020; Eastick et al., 2022a). In addition, the study on the relationship between the presence of cytoplasmic strings and the embryo viability in human *in vitro* fertilised embryos has received considerable attention. The analysis of the embryo development, using time-lapse videos, made it possible to identify and observe the behaviour of dynamically changing structures. This observation would be impossible during conventional microscopic morphology assessment. Embryologists can better understand the development and condition of early embryos by observing the appearance and behaviour of cytoplasmic strings.

Cytoplasmic strings were detected in 81.2% of the examined embryos, which is almost two times higher than the CS+ embryo ratio (43.9%) published by Ebner et al. (2020). However, the presence of CS is similar in scale to the results of Eastick et al. (2021) just in the case of the proportion of CS after conventional IVF and ICSI fertilisation, which suggests that the method of fertilisation does not affect the formation of CS (Eastick et al., 2022b).

Data did not reveal great differences neither in the early embryonic development nor in the cleavage stage morphology among the CS+ and CS- groups. However, embryos in the CS+ group developed into a higher quality blastocyst. Although it seems that other working groups who examine cytoplasmic strings do not apply traditional morphology assessment for early embryo morphology evaluation, the use of Gardner’s blastocyst morphology assessment revealed similar results (Eastick et al., 2022b).

The implantation rate was also higher in the case of CS+ embryos, but this difference was not significant. The reason is probably due to the low number of cases and the limitations of multiple pregnancy follow-ups. In addition to the limitations, a recent study examined the relationship between the cytoplasmic strings and the clinical pregnancy rate (Eastick et al., 2021). This result also supports our hypothesis that CS-containing blastocysts have a higher implantation potential.

The kinetics of early embryo development were similar within the two groups. On the other hand, the CS-containing embryos reached the blastocyst stage significantly earlier. Compared to our results, Eastick et al. (2021) found significant differences in early embryo development (PN appearance, tPNf, t2, t3, t4, t5, t6, and t8) and the time to reach blastocyst stages (Eastick et al., 2021) between embryos with and without CS. These results suggest that CS+ embryos have a better ability to develop and implant into the uterus. Based on our results, we can conclude that the early embryo development and quality were not related to the presence of CS, but the presence of cytoplasmic strings is accompanied by accelerating development and better morphology in the blastocyst stage.

KIDScore and iDAScore were significantly higher in CS+ blastocysts than CS- blastocysts. This result implies that KIDScore and iDAScore are correlated with the embryo viability (Berntsen et al., 2022; Kim, Lee, and Jun 2022; Lassen, 2022). The higher score of the CS+ embryos indicates that based on the analysis of the large IVF database, the artificial intelligence system considered the CS+ embryos most viable. To the best of our knowledge, this is the first report which shows the relationship between the morphokinetic scores (KID and iDA) and the presence of cytoplasmic string.

Time-lapse imaging allows us to follow the preimplantation embryo development including the whole cavitation process. In addition to these benefits, we were unable to detect the growth of the traversing cytoplasmic strings through the blastocoelic cavity similar to the Salas-Vidal and Lomelí’s working groups in the case of the mouse embryos (Salas-Vidal and Lomelí, 2004). This observation may indicate the fact that CS must be formed within the early stages of cavitation, and this suggestion is supported by our morphokinetic results. Cytoplasmic string formation probably occurs during compaction, and the blastulation process is responsible for their elongation and string-like structure formation in the blastocyst stage (Salas-Vidal and Lomelí, 2004; Fierro-González et al., 2013).

The observation of the morphology of the cytoplasmic string in our time-lapse images shows transport processes in both anterograde (from the inner cell mass to the mural trophectoderm cells) and retrograde (from the mTE to the ICM) directions, while Salas-Vidal and Lomelí’s groups concluded that they observed only retrograde transport in the mouse blastocyst. They also described connections between the ICM and polar trophectoderm cells in mouse blastocysts (Salas-Vidal and Lomelí, 2004). Our research group could not observe this connection, which may be due to the difference in magnitude of the time-lapse microscopes.

The observation of cytoplasmic strings in human embryos by other embryologists also suggests that bulb-like vesicle transport occurs along the strings (Ebner et al., 2020) in a bidirectional movement (antero- and retrograde) (Eastick et al., 2022b). Ebner and his colleagues also investigated the relationship between cytoplasmic extension and the blastocyst collapse, as well as the characterisation of filaments. In addition to that, this study does not focus on these phenomena, but we consider it necessary to validate these observations. This observation can raise the question whether the strings have different purposes at the blastocyst stage. However, we would like to pay more attention to vesicle transport along the cytoplasmic filaments in the future.

Transport was observed along the strings in all of our cases. We assume that there is a communication between the 2-cell population which may stimulate them to divide further or help them differentiate (Salas-Vidal and Lomelí, 2004). Although these results are preliminary observations and a deeper analysis of the vesicle-like transport was out of scope of this study, we consider it important to mention that there is a clearly visible communication which motivates us for further microscopic examination of this structure.

## Conclusion

The investigation of the importance and behaviour of cytoplasmic strings with time-lapse technology in human embryos is a new field, and there are still lots of questions to be answered: is vesicular transport taking place along the cytoplasmic strings? What kinds of molecules are transported between the cells? Which differentiated cell population (ICM or mural TE) produces signalling factors? Is the presence of cytoplasmic strings related to the quality of compaction and morula formation? Further examination of CS in embryos would be necessary. The identification of cell structures and molecules along the cytoplasmic strings could help us understand the origin, meaning, and importance of these structures in the blastocyst stage. Although the topic of cytoplasmic strings in the human blastocyst is not defined yet, more and more importance is being attached to its research, and further results are expected in the near future. Based on our results, CS were observed in most of the examined human blastocysts. The CS+ embryos developed into blastocysts faster, and their morphokinetic parameters were better. The scoring systems based on artificial intelligence also consider the CS+ blastocysts more viable, and it looks like that their implantation ability was higher.

All these results prove us that the presence of cytoplasmic strings was accompanied by good embryo viability. The examination of this phenomenon could help us select the most viable embryo in terms of implantation. Evaluation of the presence of cytoplasmic strings could be an additional morphologic feature in the future embryo grading system. These results put the following question in the spotlight: what is the certain function of the cytoplasmic strings during early embryogenesis?

## Data Availability

The raw data supporting the conclusion of this article will be made available by the authors, without undue reservation.

## Abbreviations

CS+, blastocyst with cytoplasmic strings; CS-, blastocyst without cytoplasmic strings; ET, embryo transfer; FSH, follicular stimulating hormone; GnRH, gonadotropin-releasing hormone antagonist; hCG, human chorion gonadotropin; hMG, human menopausal gonadotropin; ICM, inner cell mass; ICSI, intracytoplasmic sperm injection; iDAScore, Intelligent Data Analysis Score; IVF, *in vitro* fertilisation (treatment); KIDScore, Known Implantation Data Score; TE, trophectoderm cells; tPNf, time of the pronucleus fading; t2, t4, … tn, time taken to divide into 2, 4, … n cells; tM, time of end of compaction; tB, time of the full blastocyst stage.
